# SLR-Net: Lightweight and Accurate Detection of Weak Small Objects in Satellite Laser Ranging Imagery

**DOI:** 10.3390/s26020732

**Published:** 2026-01-22

**Authors:** Wei Zhu, Jinlong Hu, Weiming Gong, Yong Wang, Yi Zhang

**Affiliations:** 1 Institute of Seismology, China Earthquake Administration, Wuhan 430071, Chinahujinlong23@mails.ucas.ac.cn (J.H.);; 2 Hubei Key Laboratory of Earthquake Early Warning, Hubei Earthquake Agency, Wuhan 430071, China; 3 Xinjiang Astronomical Observatory, Chinese Academy of Sciences, Urumqi 830011, China; wangyong@xao.ac.cn

**Keywords:** Satellite Laser Ranging (SLR), small object detection, lightweight network, SLR-Net, feature fusion

## Abstract

To address the challenges of insufficient efficiency and accuracy in traditional detection models caused by minute target sizes, low signal-to-noise ratios (SNRs), and feature volatility in Satellite Laser Ranging (SLR) images, this paper proposes an efficient, lightweight, and high-precision detection model. The core motivation of this study is to fundamentally enhance the model’s capabilities in feature extraction, fusion, and localization for minute and blurred targets through a specifically designed network architecture and loss function, without significantly increasing the computational burden. To achieve this goal, we first design a DMS-Conv module. By employing dense sampling and channel function separation strategies, this module effectively expands the receptive field while avoiding the high computational overhead and sampling artifacts associated with traditional multi-scale methods, thereby significantly improving feature representation for faint targets. Secondly, to optimize information flow within the feature pyramid, we propose a Lightweight Upsampling Module (LUM). Integrating depthwise separable convolutions with a channel reshuffling mechanism, this module replaces traditional transposed convolutions at a minimal computational cost, facilitating more efficient multi-scale feature fusion. Finally, addressing the stringent requirements for small target localization accuracy, we introduce the MPD-IoU Loss. By incorporating the diagonal distance of bounding boxes as a geometric penalty term, this loss function provides finer and more direct spatial alignment constraints for model training, effectively boosting localization precision. Experimental results on a self-constructed real-world SLR observation dataset demonstrate that the proposed model achieves an ${\mathrm{mAP}}_{50:95}$ of 47.13% and an F1-score of 88.24%, with only 2.57 M parameters and 6.7 GFLOPs. Outperforming various mainstream lightweight detectors in the comprehensive performance of precision and recall, these results validate that our method effectively resolves the small target detection challenges in SLR scenarios while maintaining a lightweight design, exhibiting superior performance and practical value.

## 1. Introduction

Satellite Laser Ranging (SLR) is currently recognized as one of the most precise ground-based optical space geodetic techniques [1,2,3,4]. Its fundamental principle involves calculating the satellite-to-ground distance by precisely measuring the round-trip time of flight of laser pulses between a ground station and cooperative components carried by the target satellite, such as Corner Cube Retroreflectors (CCRs) or Laser Retroreflector Arrays (LRAs). SLR observational data plays an irreplaceable role in fields such as earth-satellite laser time and frequency transfer [5,6], precise orbit determination (POD) [7], the determination of global geodetic reference frames and parameters [8,9,10], and space debris monitoring [11].

The implementation of SLR technology relies on the precise tracking of target satellites. However, in actual observations, constrained by multiple factors such as ephemeris prediction errors, telescope optical axis pointing deviations, and jitter caused by atmospheric turbulence, the accuracy of blind tracking relying solely on ephemeris predictions often fails to meet requirements. Therefore, during the target’s optical visibility period (specifically during nighttime observations, excluding Earth shadow eclipses, where optical imaging is ineffective), it is typically necessary to utilize images acquired by CCD cameras for closed-loop correction to ensure the target remains in the center of the detector’s effective Field of View (FOV) in real-time. However, limited by weak echo signals, stellar interference, and complex noise environments [12] (encompassing sky background radiation, detector dark current, and readout noise), target satellites in CCD images are often submerged in strong background noise, appearing as minute, dim point targets that are extremely difficult to distinguish. Moreover, a ‘stop-and-go’ strategy to avoid laser interference is operationally infeasible, as the introduced latency fails to compensate for high-frequency pointing jitter, necessitating simultaneous detection during laser emission. Consequently, achieving precise detection of such targets has become a critical bottleneck currently constraining the development of end-to-end automated SLR.

Traditional object detection algorithms (e.g., Viola-Jones [13], HOG [14], and DPM [15]) rely on hand-crafted feature design. Constrained by shallow representation capabilities, these methods struggle to capture deep semantic information, resulting in poor robustness to noise and limited generalization performance [16]. In contrast, deep learning-based algorithms have evolved into three mainstream paradigms: the two-stage architecture (e.g., Faster R-CNN [17]) that pursues high precision via a “coarse-to-fine” strategy; the one-stage architecture (e.g., YOLO [18,19,20]) that models detection as a regression problem to balance efficiency; and the Transformer paradigm (e.g., DETR [21]) that introduces self-attention mechanisms to break the locality limitation of convolutions [22].

Benefiting from the powerful feature extraction and semantic abstraction capabilities of deep networks, the aforementioned generic detectors perform exceptionally well in related fields such as astronomical observation and remote sensing interpretation. However, directly transferring them to the SLR task yields suboptimal results. SLR-CCD images are characterized by extremely small targets (sub-pixel level) and high background noise. In such extreme scenarios, the classic downsampling mechanism in CNN architectures often leads to the irreversible loss of critical spatial details. Meanwhile, the global attention mechanism relied upon by Transformers struggles to focus on informative regions for point targets lacking texture and shape semantics, tending instead to aggregate background noise. Given the dilemma between feature preservation and noise suppression faced by existing methods, designing an efficient detection network specialized for SLR dim and small targets has become an urgent need in this field.

The remainder of this paper is organized as follows: Section 2 details the proposed SLR-Net architecture, including the DMS-Conv and LUM modules. Section 3 introduces the dataset collection and analyzes target features, followed by the experimental setup, comparative results, and ablation studies. Finally, Section 4 concludes the paper.

## 2. Method

To effectively address the unique challenges characterizing Satellite Laser Ranging (SLR) imagery—namely, tiny object sizes, low Signal-to-Noise Ratio (SNR), and feature ambiguity caused by blurring—this paper proposes a specialized, optimized, lightweight, and high-performance object detection network.

This study adopts the efficient and concise architecture of YOLOv11 [23] as the baseline framework. While retaining the design philosophy of the efficient CSPNet (Cross Stage Partial Network) backbone and the PANet (Path Aggregation Network), targeted reconstructions are implemented at key nodes of feature extraction and fusion. However, when directly applied to scenarios involving extremely small targets such as SLR, the standard configuration of YOLOv11 exhibits limitations in its standard components regarding feature extraction, multi-scale fusion, and bounding box regression.

Specifically, the main innovations of the proposed method are concentrated in the following three aspects:

1. **Feature Extraction Optimization:** To address the issue of tiny object features being easily lost in deep networks, we design novel convolution modules DMS-Conv, to enhance the network’s feature representation capabilities.
2. **Feature Fusion Enhancement:** To improve the efficiency of information flow between feature maps of different scales, we propose a more lightweight and efficient upsampling fusion mechanism, the Lightweight Upsampling Module (LUM).
3. **Localization Accuracy Improvement:** To overcome the deficiencies of traditional loss functions in bounding box regression for small targets, we introduce a new geometric constraint, MPD-IoU Loss, to guide the model toward more precise localization.

**Figure 1:**
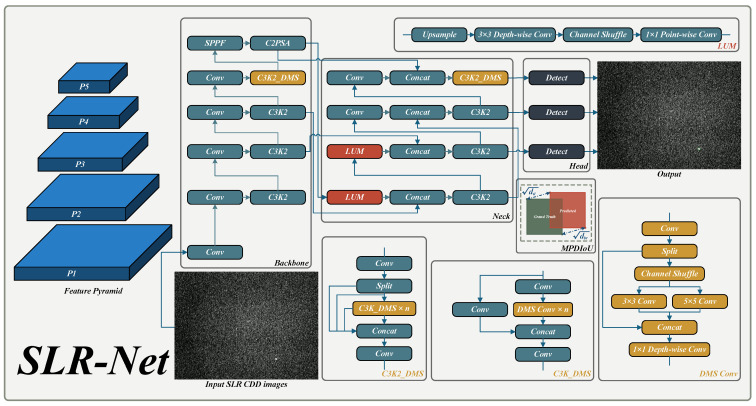
Structure of SLR-Net.

The overall architecture of the proposed SLR-Net is illustrated in Figure 1.

### 2.1. Dense Multi-Scope Convolution (DMS-Conv)

For the multi-scale, star-like small targets in SLR, expanding the receptive field to fuse contextual information is crucial during feature extraction. Common methods employ parallel multi-scale convolutions or multi-scale dilated convolutions. However, parallel multi-scale convolutions inevitably introduce high computational costs and parameter overheads. While multi-scale dilated convolutions can effectively expand the receptive field at a lower computational cost, their inherent grid effect leads to discontinuous feature sampling. For small targets in SLR, which occupy only a few pixels, it is extremely easy to completely miss the target within the sampling “holes” or destroy the integrity of their feature distribution due to discontinuous sampling, resulting in missed detections or false alarms.

**Figure 2:**
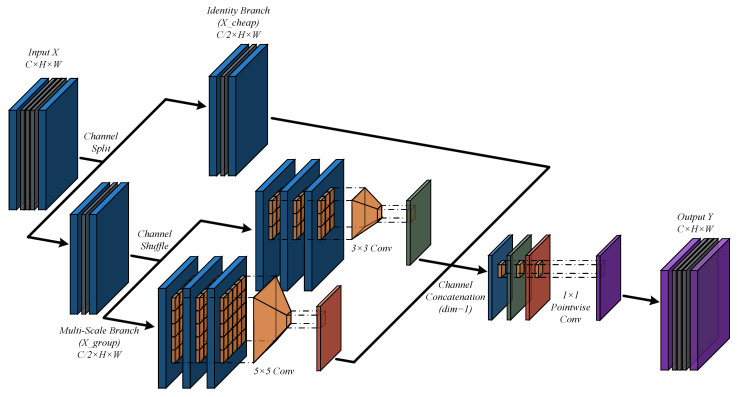
Structure of DMS-Conv.

To address this challenge, we designed an efficient **Dense Multi-Scope Convolution (DMS-Conv)**, the structure of which is illustrated in Figure 2. It aims to achieve powerful and rich feature representation with extremely low computational overhead through a Dense Sampling strategy without introducing sampling artifacts. The core idea lies in functionally allocating computational resources along the channel dimension, restricting expensive spatial convolution operations to feature subspaces, thereby capturing diverse receptive field features without introducing sampling artifacts.

Specifically, for a given input feature map $X\in R^{B\times C\times H\times W}$, DMS-Conv first splits it evenly along the channel dimension into two parallel branches: $X=\{X_{cheap},X_{group}\}$. Here, $X_{cheap}\in R^{B\times C/2\times H\times W}$ serves as an approximate identity-like mapping and is passed directly to the output to preserve the original information in the input features. The other branch, $X_{group}\in R^{B\times C/2\times H\times W}$, is used for subsequent multi-scale feature extraction, as shown in Equation (1).

We reshape $X_{group}$ along the channel dimension into *G* groups and apply a corresponding spatial convolution $F_{k_{g}}$ from a preset kernel set $K=\{k_{1},\ldots ,k_{g}\}$ to each group. To maximize efficiency, the lightweight spatial convolution within each group is performed on a compressed channel width min_ch (experimentally set to min_ch≥16). The outputs of all groups ${\left\{Y_{g}\right\}}_{g=1}^{G}$ are concatenated along the channel dimension to form $Y_{group}$, as shown in Equation (2). Finally, we concatenate the output of the Cheap Branch ($X_{cheap}$) with the output of the multi-scale path ($Y_{group}$) and perform cross-channel information remixing through an efficient 1×1 convolution (linear projection) to obtain the final output *Y*:

(1) $$X_{cheap},X_{group}=\mathrm{Split}\left(X\right)$$

(2) $$Y_{group}=\mathrm{Concat}{\left\{{\mathrm{Conv}}_{k_{i}}\left(X_{group_{i}}\right)\right\}}_{i=1}^{G}$$

(3) $$Y={\mathrm{Conv}}_{1\times 1}\left(\mathrm{Concat}\left(X_{cheap},Y_{group}\right)\right),\;Y\in R^{B\times C\times H\times W}$$

This design strictly limits the computationally intensive k×k convolutions to the feature subspace while utilizing 1×1 convolutions to restore full-channel information interaction. DMS-Conv not only effectively expands the receptive field with minimal computational overhead but also enhances feature diversity and expressiveness, forming a richer representation. In the network design, we apply DMS-Conv within the bottleneck structure with channel reduction to maximize its efficiency and performance gains.

### 2.2. Lightweight Upsampling Module (LUM)

**Figure 3:**
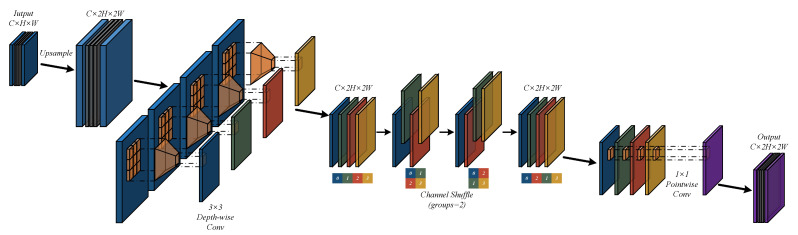
Structure of LUM.

In the neck structure of the detector, the upsampling operation is responsible for restoring high-level semantic features to a higher resolution for fusion with low-level detailed features. However, traditional upsampling methods, such as Transposed Convolution which incurs huge computational overheads, often become a bottleneck in lightweight model design. To construct a more efficient and powerful feature fusion path, we designed and proposed a novel **Lightweight Upsampling Module (LUM)**, the structure of which is illustrated in Figure 3. It aims to replace traditional upsampling layers in a lightweight manner to achieve a balance between efficiency and performance.

Specifically, for a given deep input feature map $X_{in}\in R^{B\times C\times H\times W}$, the module first doubles its spatial dimensions via bilinear interpolation and immediately applies a Depthwise Separable Convolution (DWC) for preliminary spatial feature extraction, as shown in Equation (4). Since depthwise convolution operates independently per channel, to break the limitation of isolated channel information, we subsequently introduce a Channel Shuffle mechanism. Drawing on the idea of ShuffleNet, this operation breaks the independence between channels through efficient and uniform rearrangement, laying a more efficient feature foundation for subsequent cross-channel information fusion, as shown in Equation (5). Finally, we utilize a 1×1 Pointwise Convolution (PWC) to perform a weighted combination of the shuffled features, achieving cross-channel information fusion while generating the final upsampled output $Y\in R^{B\times C\times 2H\times 2W}$. The entire process can be represented by the following sequence of equations:

(4) $$X^{\prime }=F_{DWC}\left(\mathrm{Upsample}\left(X_{in}\right)\right)$$

(5) $$X^{\prime \prime }=\mathrm{ChannelShuffle}\left(X^{\prime }\right)$$

(6) $$Y=F_{PWC}\left(X^{\prime \prime }\right)$$

In summary, the LUM module constructs an efficient and powerful upsampling unit by ingeniously combining upsampling, depthwise separable convolution, channel shuffle, and pointwise convolution. It not only significantly reduces the computational burden of the upsampling path but also enhances the cross-channel communication capability of features through the introduction of the channel shuffle mechanism, contributing to the quality of multi-scale feature fusion and thereby improving the detection performance for small-sized targets.

### 2.3. MPD-IoU Loss

Among existing IoU-series loss functions, CIoU has been widely used for bounding box regression in object detection. It introduces center point distance penalties and aspect ratio constraints on top of the traditional IoU, improving localization accuracy to a certain extent. However, CIoU still has deficiencies in its constraint mechanism: its aspect ratio term contributes limitedly to small object scenarios, and the single-point metric of center distance struggles to fully characterize the alignment differences between the predicted box and the ground truth box at the boundaries.

**Figure 4:**
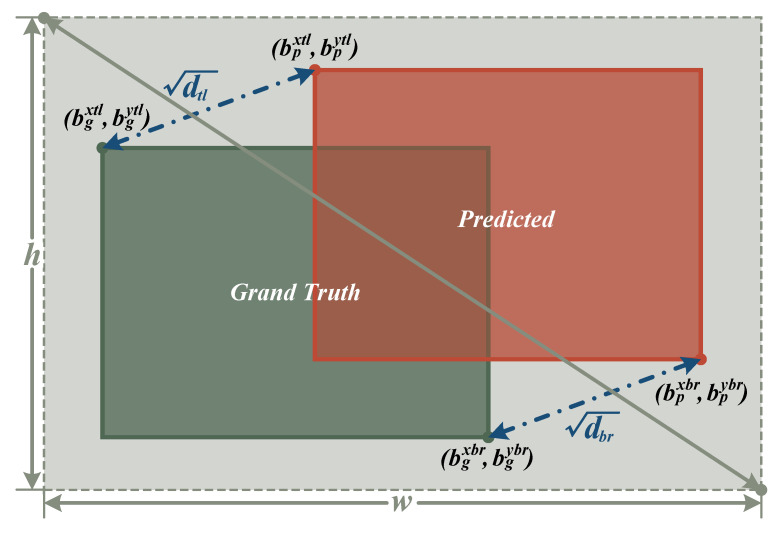
Schematic diagram of MPD-IoU.

Therefore, this paper introduces a new loss function—**MPD-IoU**, whose schematic diagram is shown in Figure 4. Its core idea is to further introduce the Euclidean distances between two sets of diagonal points of the predicted box and the ground truth box as geometric penalties on top of the IoU calculation, thereby describing the spatial consistency of the bounding boxes in greater detail. Specifically, let the predicted box and the ground truth box be $\left(b_{p}^{xtl},b_{p}^{ytl},b_{p}^{xbr},b_{p}^{ybr}\right)$ and $\left(b_{g}^{xtl},b_{g}^{ytl},b_{g}^{xbr},b_{g}^{ybr}\right)$ respectively; MPD-IoU is defined as:

(7) $$\mathrm{MPDIoU}=\mathrm{IoU}-\frac{d_{tl}}{S}-\frac{d_{br}}{S}$$

where IoU represents the Intersection over Union of the two boxes. $d_{tl}=\sqrt{{\left(b_{g}^{xtl}-b_{p}^{xtl}\right)}^{2}+{\left(b_{g}^{ytl}-b_{p}^{ytl}\right)}^{2}}$ is the Euclidean distance of the top-left corner points, and $d_{br}=\sqrt{{{(}_{g}^{xbr}-b_{p}^{xbr})}^{2}+{\left(b_{g}^{ybr}-b_{p}^{ybr}\right)}^{2}}$ is the Euclidean distance of the bottom-right corner points. *S* is a normalization factor to control the magnitude of the penalty.

Compared with CIoU, MPD-IoU possesses the following advantages:

1. **Enhanced Boundary Alignment Constraint:** By simultaneously considering the registration degree of both the top-left and bottom-right corners, MPD-IoU achieves a finer-grained alignment metric at the geometric level compared to a single center point.
2. **Adaptation to Small Object Detection:** In scenarios with star-like small targets in SLR, aspect ratio differences contribute limitedly to regression, whereas the deviation of boundary points directly determines whether the target is covered. Thus, MPD-IoU fits the task requirements better.
3. **Optimized Convergence Stability:** Corner distance constraints provide clearer gradient information, enabling the model to converge faster to high-quality bounding box predictions during training.

In summary, MPD-IoU maintains the efficiency of CIoU while further reinforcing the spatial geometric constraints of the bounding box, making it particularly suitable for high-precision localization tasks such as low-SNR, star-like small object detection.

## 3. Experiments

### 3.1. Dataset

The optimization of deep learning model performance relies heavily on high-quality training data. Addressing the scarcity of CCD image datasets in the Satellite Laser Ranging (SLR) field, this study conducted data collection in the actual operating environment of the TROS1000 [24] system. TROS1000 is the world’s largest aperture mobile SLR system developed by the Institute of Seismology, China Earthquake Administration. It is equipped with a 1-m aperture optical telescope with a maximum range of 36,000 km and is deployed at the Nanshan Observation Station of Xinjiang Astronomical Observatory, Chinese Academy of Sciences.

**Figure 5:**
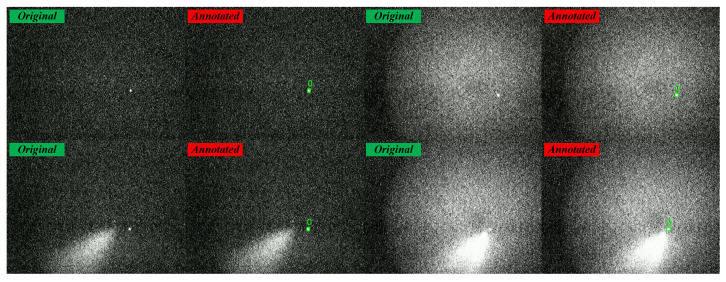
Visualization of typical samples from the dataset.

The dataset was collected from CCD cameras at the ground-based SLR station, covering real observation data under different nights and atmospheric conditions. We used professional annotation tools to precisely label image frames containing satellite laser reflection signals and synchronously labeled non-ranging targets within the field of view. This annotation strategy not only helps accurately screen target candidate regions during SLR observations but also provides data support for multi-task starry sky background detection. Ultimately, the dataset contains 1156 images with 2162 valid target instances. The entire dataset was divided into training, validation, and test sets in a ratio of 7:2:1. Typical samples from the constructed dataset are visualized in Figure 5.

### 3.2. Dataset Feature Analysis

**Figure 6:**
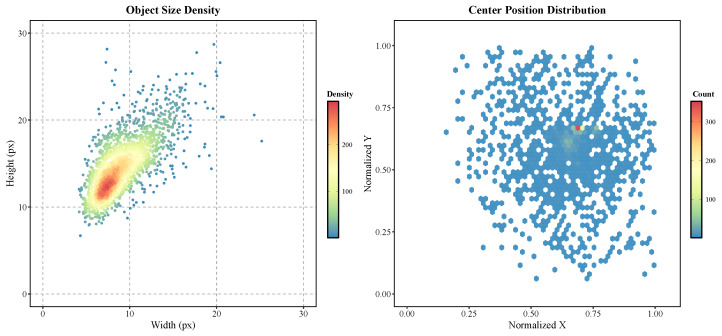
Spatial and scale distribution analysis of the ground truth bounding boxes.

After constructing the dataset, we systematically analyzed the target features. We first statistically analyzed the center positions of all targets in the dataset and plotted a spatial distribution heatmap (Figure 6). It is evident from the figure that the target distribution is not uniformly random but exhibits significant **center aggregation characteristics**. The vast majority of target instances are concentrated in the center of the image and its vicinity, particularly in the normalized coordinate range of (0.6, 0.6) to (0.8, 0.7), where target density is highest. This distribution characteristic is highly correlated with the automatic tracking task of the SLR system, which strives to maintain the target under test at the center of the field of view.

Target size is also a key factor determining detection difficulty. As shown in Figure 6, we visualized the pixel dimensions (width and height) of all target instances. The scatter plot clearly reveals the core challenge of this dataset: **target sizes are generally extremely small**. Widths are concentrated between 5 and 15 pixels, and heights between 5 and 20 pixels. The size distribution of the entire dataset forms a dense cluster in the bottom-left corner of the chart, with only a very few large outliers. This typical tiny target distribution characteristic poses a significant test to the detection capability of the model.

### 3.3. Experimental Environment and Evaluation Metrics

The experimental environment configuration is shown in Table 1.

The training parameters were set as follows: training duration of 100 epochs, batch size of 16, and image size of 640×640. The model uses the SGD optimizer for parameter optimization, with an initial learning rate of 0.01 and a momentum parameter of 0.937. To prevent overfitting, a weight decay strategy was adopted with a value of $5\times 10^{-4}$.

To comprehensively evaluate the model’s effectiveness, Precision (*P*), Recall (*R*), ${\mathrm{mAP}}_{50}$, ${\mathrm{mAP}}_{75}$, ${\mathrm{mAP}}_{50:95}$, and F1-Score were selected as metrics. Additionally, Params and GFLOPs were used to compare model parameters and running speed.

The metrics are defined as follows:

- **Precision (***P***):**
  $$P=\frac{TP}{TP+FP},$$
  where TP is True Positives and FP is False Positives.
- **Recall (***R***):**
  $$R=\frac{TP}{TP+FN},$$
  where FN is False Negatives.
- **F1-Score:**
  $$F1=2\times \frac{P\times R}{P+R}.$$
- **Mean Average Precision (mAP):** For a given IoU threshold *t*,
  $${\mathrm{AP}}_{t}=\int_{0}^{1}\mathrm{precision}\left(r\right)\;dr,$$
  and
  $${\mathrm{mAP}}_{t}=\frac{1}{C}\sum_{c=1}^{C}AP_{t,c}.$$
- ${\mathrm{mAP}}_{50:95}$**:**
  $${\mathrm{mAP}}_{50:95}=\frac{1}{10}\sum_{i=0}^{9}{\mathrm{mAP}}_{0.50+0.05i}.$$

### 3.4. Ablation Experiments

We conducted extensive ablation studies to systematically verify the effectiveness and efficiency of the innovative components proposed in this paper—**DMS-Conv**, **Lightweight Upsampling Module (LUM)**, and **MPD-IoU Loss**. All experiments were performed on the SLR dataset described in Section 3.5.2, and results are summarized in Table 2.

**Regarding the Architectural Component Analysis**, we first validated the performance of the DMS-Conv and LUM components. It was found that integrating either module individually did not show an overwhelming advantage in core metrics measuring localization accuracy. However, when both modules were integrated, we observed significant synergistic gains: the model’s ${\mathrm{mAP}}_{50}$ and Recall reached globally optimal levels. This clearly indicates a strong complementarity between the powerful feature extraction capability of DMS-Conv and the efficient feature fusion path of LUM. Their combination enables the model to “see clearer and find more completely” from strong noise.

**In the Loss Function Analysis**, based on the optimal architecture, the introduction of the MPD-IoU loss function achieved a significant breakthrough in core localization accuracy metrics, especially under stricter evaluation standards like ${\mathrm{mAP}}_{50:95}$ and ${\mathrm{mAP}}_{75}$. Precision and F1-Score also reached their global best. This result strongly confirms that while the optimized architecture gives the model the ability to “see clearly”, the advanced MPD-IoU loss teaches it how to “draw accurately”. For SLR targets with blurred edges, the direct geometric constraints provided by MPD-IoU are key to high-precision localization.

**As for the Model Complexity Analysis**, Table 2 also reports the complexity and computational overhead. From the baseline to our final complete model, Parameters decreased slightly from 2.58 M to 2.57 M, while GFLOPs increased slightly from 6.3 to 6.7. This indicates that the significant performance improvement comes from superior, problem-specific architectural design rather than simply increasing parameters.

### 3.5. Comparative Experiments

To validate the effectiveness of SLR-Net, we conducted comparative experiments against 15 mainstream object detectors. The benchmarks encompass classical two-stage algorithms (e.g., Faster R-CNN [17]), the YOLO series [20,23,26,27,28,29,30,31], and detectors representing the Transformer paradigm (e.g., DINO [32]). Detailed comparative data are presented in Table 3.

#### 3.5.1. Results and Analysis

**In terms of detection accuracy**, our model achieved an ${\mathrm{mAP}}_{50}$ of **92.36%** and an F1-Score of **88.24%**. With a comparable parameter count (2.57 M), its ${\mathrm{mAP}}_{50}$ surpassed YOLOv10-n (+3.81%) and YOLOv8-n (+10.71%), respectively, and also exceeded the medium-scale network YOLOv5-m (25.05 M). The data indicate that the feature enhancement module tailored for point targets effectively overcomes the bottleneck of lightweight networks in extracting weak features.

**In terms of model adaptability**, the experiments revealed that several large-scale general-purpose detectors exhibited suboptimal performance on this specific task. Traditional two-stage algorithms showed limited accuracy; even DINO, a benchmark model based on the Transformer architecture with 47.54 M parameters, achieved an ${\mathrm{mAP}}_{50}$ of **87.70%**, which is lower than that of our model. This phenomenon may be attributed to the fact that excessively deep layers or global attention mechanisms, when processing point targets lacking semantic information, are more prone to introducing background noise interference, thereby constraining detection performance.

**Regarding inference efficiency and limitations**, the model achieved an inference speed of **130.39 FPS** on a single GPU (2.57 M Params/6.70 GFLOPs). It is worth noting that the computational modules introduced to enhance the capture of weak targets imposed a certain inference burden, resulting in a decrease in speed compared to the **Base** model (201.28 FPS) and some minimalist models (e.g., YOLOv3-tiny). However, considering the stringent accuracy requirements of SLR systems and the fact that **130 FPS** far exceeds the real-time processing standard (>30 FPS), this strategy of trading a minor speed loss for significant accuracy gains (+1.45% in ${\mathrm{mAP}}_{50}$) is considered acceptable and efficient for practical deployment.

To rigorously validate the effectiveness and stability of the proposed method, we compared SLR-Net with the YOLOv11 baseline across five independent experimental runs to account for stochastic fluctuations. Table 4 reports the mean and standard deviation for key metrics. As shown, SLR-Net achieves consistent improvements across all indicators. While the improvement in general detection (${\mathrm{mAP}}_{50}$) is steady (+0.90%), the most significant gain is observed in strict localization accuracy (${\mathrm{mAP}}_{75}$), which increases by **3.88%** (from 40.02% to 43.90%). In the context of Satellite Laser Ranging (SLR), the telescope servo system relies on precise centroid coordinates to maintain stable tracking; a “loose” detection (low IoU) can induce jitter. Therefore, this substantial boost in high-IoU performance demonstrates that the proposed MPD-IoU Loss and DMS-Conv significantly refine bounding box regression, transforming “rough detection” into the “high-precision localization” required for automated observations.

#### 3.5.2. Visualization Analysis

**Figure 7:**
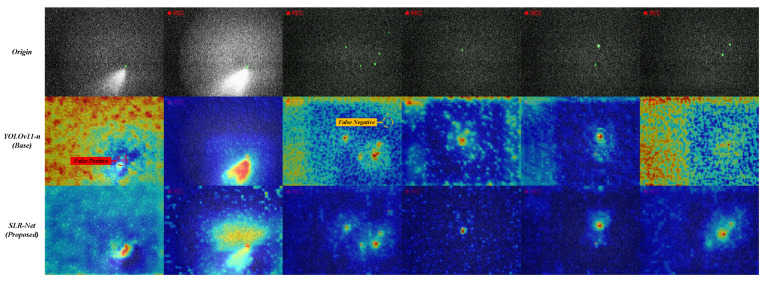
Visual comparison of response heatmaps between baseline models and SLR-Net.

**Figure 8:**
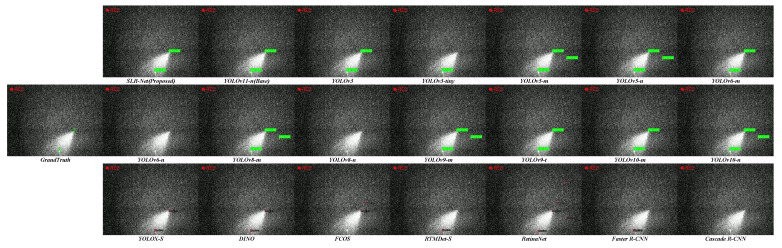
Qualitative detection results on challenging SLR scenes with different methods.

To further qualitatively analyze the detection behavior of different models, we provide visualization results including response heatmaps and final detection outputs, as shown in Figure 7 and Figure 8. These visualizations are generated on representative SLR scenes with strong background clutter, varying noise levels, and extremely small targets.

The heatmap comparisons (Figure 7) reveal that mainstream detectors tend to activate strongly on high-intensity background regions or structured noise, which often leads to false positives. In contrast, the proposed SLR-Net produces more compact and target-centered responses, with suppressed background activation. This indicates that SLR-Net is able to better capture discriminative cues of weak targets while mitigating interference from complex background patterns.

In terms of detection results (Figure 8), several challenging cases are illustrated, including sparse star-like targets and low-contrast targets embedded in clutter. As highlighted in the examples, baseline models either miss the target (false negatives) or incorrectly respond to background artifacts (false positives). Benefiting from its enhanced feature representation and balanced precision–recall behavior, SLR-Net successfully detects these targets with accurate localization and reduced false alarms.

Overall, the visualization results are consistent with the quantitative evaluations in Table 3, demonstrating that the proposed model not only achieves competitive performance in terms of metrics, but also exhibits superior robustness and reliability in real SLR detection scenarios.

## 4. Conclusions

To address the challenges associated with Satellite Laser Ranging (SLR) imagery specifically, minute target sizes, low signal-to-noise ratios (SNRs), and susceptibility to feature loss—this paper proposes an efficient and lightweight detection network named SLR-Net. The network innovatively incorporates the DMS-Conv module, which effectively enhances feature extraction capabilities and expands the receptive field by employing dense sampling and channel separation strategies. Simultaneously, the Lightweight Upsampling Module (LUM) is utilized to optimize multi-scale feature fusion, and in conjunction with the MPD-IoU loss function, the localization accuracy for minute targets is significantly improved.

Experimental results demonstrate that SLR-Net achieves superior performance on real-world SLR datasets. With only 2.57 M parameters, it attains an ${\mathrm{mAP}}_{50:95}$ of 47.13%, significantly outperforming current mainstream lightweight detectors while maintaining extremely low computational costs. This study not only validates the practical value of the proposed method in automated SLR observation systems but also provides a solid foundation for future real-time deployment on edge computing devices. Future work will focus on further expanding the dataset and exploring the generalization capability of the model in more complex environments.

## Figures and Tables

**Table 1:** Experimental Environment Configuration.

Item	Specification
Operating System	Ubuntu 22.04
GPU	RTX 4090 (24 GB)
CPU	16 vCPU Intel (Santa Clara, CA, USA) Xeon(R) Platinum 8352V CPU @ 2.10 GHz
Memory	120 GB
Programming Language	Python 3.10
Framework	PyTorch 2.1.0 + CUDA 12.1
IDE	JupyterLab

**Table 2:** Ablation study of SLR-Net on the SLR dataset. **DMS**: DMS-Conv, **LUM**: Lightweight Upsampling Module, **MPD**: MPD-IoU loss [25]. Best results are shown in **bold**, and second-best are underlined.

Model	Components			Complexity		Performance (%)					
	DMS	LUM	MPD	Params (M)	GFLOPs	Prec.	Recall	F1	${\mathrm{mAP}}_{50}$	${\mathrm{mAP}}_{75}$	${\mathrm{mAP}}_{50:95}$
Baseline				2.58	**6.30**	88.51	**86.89**	87.69	90.91	42.65	**47.24**
+ LUM		✓		2.59	6.30	88.87	86.77	87.80	91.20	42.70	47.22
+ DMS	✓			**2.56**	6.20	89.23	86.64	87.91	91.49	42.76	47.20
+ DMS + LUM	✓	✓		2.57	6.70	89.94	86.39	88.13	92.07	42.87	47.15
**SLR-Net (Proposed)**	✓	✓	✓	2.57	6.70	**90.30**	86.27	**88.24**	**92.36**	**42.92**	47.13

**Table 3:** Comparison with state-of-the-art detectors on the SLR dataset. Best results are shown in **bold**, and second-best are underlined.

Model	Params (M)	GFLOPs	Prec. (%)	Recall (%)	F1 (%)	${\mathrm{mAP}}_{50}$ (%)	${\mathrm{mAP}}_{75}$ (%)	${\mathrm{mAP}}_{50:95}$ (%)	FPS
**SLR-Net (Proposed)**	2.57	6.70	90.30	86.27	**88.24**	**92.36**	42.92	47.13	130.39
Base	2.58	**6.30**	88.51	86.89	87.69	90.91	42.65	47.24	201.28
**YOLO-based Detectors**									
YOLOv3 [20]	103.67	282.20	89.57	84.15	86.77	89.45	40.11	46.93	121.94
YOLOv3-tiny [20]	12.13	18.90	79.17	37.25	50.67	59.26	14.16	24.92	275.76
YOLOv5-m	25.05	64.00	87.85	85.29	86.55	91.41	43.64	47.27	176.75
YOLOv5-n	2.50	7.10	86.98	85.11	86.03	87.44	36.50	44.11	216.34
YOLOv6-m [27]	51.98	161.10	84.30	77.45	80.73	83.36	23.76	38.00	195.63
YOLOv6-n [27]	4.23	11.80	70.90	**88.24**	78.63	82.79	19.21	35.99	221.42
YOLOv8-m [29]	25.84	78.70	86.45	87.25	86.85	91.35	42.57	48.22	195.79
YOLOv8-n [29]	3.01	8.10	81.85	83.82	82.83	81.65	30.83	38.90	211.24
YOLOv9-m [30]	20.16	77.00	86.32	86.63	86.48	90.94	40.08	46.50	124.36
YOLOv9-t [30]	**1.97**	7.60	87.90	87.75	87.82	90.94	43.34	47.35	137.14
YOLOv10-m [31]	15.31	58.90	84.35	79.90	82.07	89.54	**46.06**	**48.43**	161.16
YOLOv10-n [31]	2.27	6.50	86.31	80.37	83.23	88.55	38.24	45.63	205.11
**Anchor-Free/Transformer-based Detectors**									
YOLOX-S [33]	8.94	8.52	93.37	82.84	87.79	85.80	27.80	39.50	120.35
DINO [32]	47.54	80.70	85.37	85.78	85.57	87.70	25.60	38.50	39.95
FCOS [34]	32.11	50.29	**97.50**	38.24	54.93	78.90	20.30	32.00	100.87
RTMDet-S [35]	8.86	9.44	89.94	70.10	78.79	79.70	16.70	30.80	106.15
RetinaNet [36]	36.33	52.20	52.53	76.47	62.28	60.80	3.30	20.30	99.63
Faster R-CNN [17]	41.35	63.18	74.36	14.22	23.87	18.50	2.20	5.70	91.12
Cascade R-CNN [37]	69.15	90.98	33.33	1.47	2.82	3.10	0.00	0.60	68.43

**Table 4:** Statistical Performance Comparison. Evaluation of stability and accuracy between YOLOv11 and SLR-Net across 5 independent runs (N=5). The results (Mean ± Std) demonstrate significant improvements in high-precision localization metrics (${\mathrm{mAP}}_{75}$).

Metric	YOLOv11 (Base)	SLR-Net (Proposed)	Δ Mean
${\mathrm{mAP}}_{50}$ (%)	92.40±1.25	93.30±0.71	+0.90
${\mathrm{mAP}}_{50:95}$ (%)	46.20±0.99	47.78±0.87	+1.58
${\mathrm{mAP}}_{75}$ **(%)**	40.02±3.23	43.90±2.55	**+3.88**
Precision (%)	90.00±1.80	90.64±1.19	+0.64
Recall (%)	86.48±1.59	87.50±1.58	+1.02
F1-Score (%)	88.18±0.73	89.02±0.63	+0.84

## Data Availability

The data presented in this study are available on request from the corresponding author.
